# Stress, Anxiety, Depression, and Digital Fatigue Among Nursing Students: A Cross-Sectional Study

**DOI:** 10.3390/healthcare14131950

**Published:** 2026-07-01

**Authors:** Aslıhan Öztürk Eyimaya, Mahsa Tamaddon, Tufan Aslı Sezer, Ayfer Tezel

**Affiliations:** Department of Nursing, Faculty of Nursing, Ankara University, Ankara 06230, Türkiye; tamaddon.mahsa@gmail.com (M.T.); tasezer@ankara.edu.tr (T.A.S.); atezel@ankara.edu.tr (A.T.)

**Keywords:** digital fatigue, depression, anxiety, nursing students, digital well-being

## Abstract

**Highlights:**

**What are the main findings?**

- Nursing students reported elevated depression, anxiety, and stress alongside moderate digital fatigue.
- Specific sub-dimensions of digital fatigue—namely digital addiction, psychosomatic problems, and physical–mental fatigue—were significant predictors of these psychological symptoms, particularly among female students and heavy internet users.

**What are the implications of the main findings?**

- The findings highlight digital burden as a crucial risk factor for psychological distress, emphasizing the need for targeted interventions for future healthcare professionals.
- Integrating digital well-being strategies and screen-time management into nursing education is essential to safeguard students’ overall mental health.

**Abstract:**

**Purpose:** This study aimed to determine levels of stress, anxiety, depression and digital fatigue among nursing students and to examine the associations between digital fatigue components and these psychological outcomes. **Methods:** This descriptive and correlational cross-sectional study was conducted with 543 nursing students from a nursing faculty in Türkiye in June 2025. Data were collected face-to-face using a Personal Information Form, the Digital Fatigue Scale (DFS) and the Depression Anxiety Stress Scale-21 (DASS-21). Descriptive statistics, *t*-test, Mann–Whitney U, ANOVA, Kruskal–Wallis, correlation, and multiple regression analyses were performed. **Results:** Students reported elevated levels of depression, anxiety and stress, and a moderate level of digital fatigue (DFS total mean = 2.82 ± 0.68). Female students had significantly higher anxiety, stress and digital fatigue scores than males. Daily internet use of ≥6 h was associated with higher depression, stress and digital fatigue. DASS-21 total scores were positively correlated with DFS total and all subscales. In multivariate models, digital addiction (β = 0.178), online pressure (β = 0.104), and psychosomatic problems (β = 0.174) significantly predicted depression. Psychosomatic problems (β = 0.236) and physical–mental fatigue (β = 0.156) predicted anxiety, while digital addiction (β = 0.180), psychosomatic problems (β = 0.210) and physical–mental fatigue (β = 0.157) predicted stress. These models explained 16.2%, 17.6%, and 20.1% of the total variance, respectively (all *p* < 0.05). **Conclusions:** Digital fatigue is positively associated with depression, anxiety, and stress in nursing students. High daily internet use and female gender relate to higher symptom and fatigue scores. Incorporating digital well-being, screen-time management, and mental health support into curricula, along with institutional strategies to reduce digital burden, may protect future nurses’ psychological well-being.

## 1. Introduction

With digital technologies becoming an indispensable part of everyday life, individuals increasingly conduct work, communication, education, and leisure activities through digital platforms, which in turn markedly elevates the risk of digital fatigue [1]. In particular, the widespread use of smartphones and the tendency to remain continuously connected via social media and other online platforms may tax individuals’ cognitive and emotional resources, rendering digital fatigue a salient topic of inquiry. The literature indicates significant associations between digital fatigue and reduced cognitive performance, sleep disturbances, and heightened stress levels [1,2,3]. In addition, multiple studies have reported that problematic smartphone and internet use among university students is associated with depression, anxiety, and sleep problems [4,5,6].

With the speed and accessibility afforded by digital technologies, substantial convenience has been achieved in areas such as education and healthcare; however, these advances have also given rise to emerging risk domains, including digital addiction and digital fatigue [7,8]. Digital fatigue has been defined as a state of mental and emotional exhaustion stemming from information overload, uninterrupted notifications, expectations of constant online availability, and the demands of multitasking; it may foster a sense of “persistent arousal,” feelings of inadequacy, and an increased psychological burden [9,10]. Continuous prompts from sources such as social media, email, and news feeds can generate stress, perceived inadequacy, and psychological exhaustion, whereas prolonged screen exposure may also adversely affect physical health [11]. Physical manifestations include eye strain and headaches, neck pain, chronic fatigue, and sleep disturbances, while psychological symptoms may present as concentration difficulties, forgetfulness, anxiety, depression, and irritability. In addition, social isolation, loss of motivation, and feelings of being overwhelmed are among the commonly reported consequences of digital fatigue [12].

Digital fatigue, a state of exhaustion manifested through physical and psychological symptoms amid the increasing professional and personal use of digital devices, has become a critical concern particularly within health-related educational programs where high cognitive and emotional demands converge. Nursing education encompasses intensive theoretical coursework, clinical practicums, and an increasingly blended curriculum with online/digital components, thereby amplifying students’ exposure to digital tools as well as their academic and emotional burden. Nursing students are required to manage a multidimensional training process simultaneously—including theoretical classes, clinical practice, assignments, online courses, and clinical simulations—and consequently rely heavily on digital devices and platforms. Accordingly, assessing nursing students’ levels of stress, anxiety, depression, and digital fatigue is essential not only to safeguard their psychological well-being but also to support healthcare efficiency and patient safety [13,14].

Recent studies have demonstrated that the prevalence of depression and anxiety among nursing students may be higher than in the general university population, and that these conditions can adversely affect academic achievement, clinical performance, and professional identity development [13,15]. Problematic smartphone use and internet addiction among university students have been found to be significantly associated with sleep quality, depression, and anxiety; in particular, increased screen time during late-night hours has been reported to exacerbate sleep disturbances and psychological symptoms [4,5,6]. These findings suggest that symptoms related to digital fatigue may exert multifaceted effects on mental health and quality of life. Although the existing literature has established associations between problematic digital use and depression, anxiety, stress, and sleep problems in university students [4,5,6], studies examining the components of digital fatigue alongside depression, anxiety, and stress levels in nursing students—particularly within multivariate frameworks—remain limited. Notably, the scarcity of research testing the contribution of digital fatigue sub-dimensions (digital addiction, online pressure, physical–mental fatigue, and psychosomatic problems) to depressive symptoms, anxiety, and stress levels in nursing students underscores a significant gap in the field.

A more detailed evaluation of the relationships between digital fatigue and depression, anxiety, and stress is crucial both for safeguarding students’ mental well-being and for sustaining the professional functioning of future nurses. In this context, studies that clarify the extent to which the sub-dimensions of digital fatigue are associated with levels of depression, anxiety, and stress may inform the planning of preventive and protective interventions at the individual level and support the development of strategies within educational programs to promote digital health, digital balance, and mental well-being. The theoretical underpinning of this study is grounded in the Technostress Framework and Cognitive Overload Theory. Technostress, particularly the ‘techno-exhaustion’ component, suggests that prolonged exposure to digital demands exceeds an individual’s cognitive resources, leading to psychological strain. While existing literature has established a general link between technology use and stress, there is a lack of granular evidence regarding which specific dimensions of digital fatigue—mental, physical, or psychosomatic—are the primary drivers of distress in nursing students. This study advances the literature by dissecting these sub-dimensions to identify targeted areas for institutional intervention.

In Türkiye, nursing education is a four-year undergraduate program. Attendance in both theoretical classes and clinical practicums is mandatory, which contributes to the high workload and potential digital burden as students use digital platforms for mandatory course management systems. This study aimed to determine nursing students’ levels of digital fatigue, depression, anxiety, and stress and to examine the relationship between these variables.

### Research Hypotheses

Within the scope of this study, the following hypotheses were formulated:

**H1.**  *There is a significant positive association between digital fatigue and levels of depression, anxiety, and stress.*

**H2.**  *The sub-dimensions of digital fatigue significantly predict depression, anxiety, and stress levels among nursing students.*

## 2. Method

### 2.1. Design

The study was designed as a descriptive, correlational cross-sectional investigation. The reporting and methodological structure were guided by the Strengthening the Reporting of Observational Studies in Epidemiology (STROBE) statement for observational research (File S1).

### 2.2. Participants and Sample Size

The study was conducted at Ankara University Faculty of Nursing, one of the oldest and most established nursing schools in Türkiye. The faculty provides a rigorous four-year undergraduate program located in the capital city, where students are exposed to high academic standards and diverse clinical practice environments. The study population consisted of 838 students enrolled in the Nursing Department of a university in Türkiye. To determine an appropriate sample size, a sample size calculation formula for situations in which the population size is known was applied [16].

$$n=\frac{\left(N*t^{2}*p*q\right)}{\left(\left[d^{2}*\left(N-1\right)\right]+\left[t^{2}*p*q\right]\right)}$$

N: Population size (838);

n: Sample size to be determined;

p: Estimated proportion of the attribute present in the population (0.50);

q: Estimated proportion of the attribute absent in the population (0.50);

t: Theoretical value from the t-distribution table at a specified confidence level and degrees of freedom (1.96);

d: Desired margin of error (±deviation) based on the estimated proportion (0.05).

Based on this calculation, a minimum sample of 263 students was deemed sufficient for a population of 838. The gender distribution of the participants (89% female) is consistent with the overall student profile of the nursing faculty and reflects the traditional demographic trends in the nursing profession in Türkiye.

### 2.3. Data Collection

Data were collected in person in June 2025. The questionnaires were administered face-to-face in classroom settings during students’ breaks and at times convenient for them; completion took approximately 15 min per student. Participants were informed about the aim of the study, and participation was strictly voluntary. Students were asked to respond without any obligation or linkage to course grades or academic evaluation. Of the 838 eligible students, 543 completed the questionnaire, yielding a response rate of approximately 65%.

Inclusion criteria were voluntary participation and the absence of any sensory or cognitive impairments (e.g., hearing, visual, or comprehension difficulties) that could hinder the completion of the data collection forms. Exclusion criteria were: (1) students who were on leave or not actively attending classes during the data collection period, (2) students who had a diagnosed chronic psychiatric disorder that might independently affect DASS-21 scores, and (3) students who submitted incomplete questionnaires.

### 2.4. Data Collection Instruments

Data were collected using a Personal Information Form, the Digital Fatigue Scale (DFS), and the 21-item Depression, Anxiety, and Stress Scale (DASS-21).

#### 2.4.1. Personal Information Form

The personal information form was developed by the researchers based on a review of the relevant literature. It consisted of items querying sociodemographic characteristics and technology usage patterns hypothesized to influence students’ levels of digital fatigue, depression, anxiety, and stress. The form included questions regarding age, gender, grade level, income status, average daily internet usage duration, ownership of technological devices, the most frequently used digital tools, and the primary purposes for using these digital platforms.

#### 2.4.2. Digital Fatigue Scale (DFS)

The Digital Fatigue Scale (DFS), developed by Tutar and Mutlu (2024) [14] to assess perceived digital fatigue among individuals who use digital technology intensively, is a 28-item instrument rated on a five-point Likert scale (1 = strongly disagree, 5 = strongly agree). The DFS is an original scale developed in Turkish and aims to assess digital fatigue multidimensionally among individuals with high levels of digital technology use [14]. It comprises four factors: Digital Addiction, Online Pressure, Physical–Mental Fatigue, and Psychosomatic Problems. In the scale development study, the overall Cronbach’s alpha coefficient was reported as 0.95; subscale Cronbach’s alpha coefficients were 0.94 for Digital Addiction, 0.90 for Online Pressure, 0.88 for Physical–Mental Fatigue, and 0.79 for Psychosomatic Problems. For scoring, mean scores are calculated for each subscale, and the overall mean score reflects the level of digital fatigue. Mean scores of 4–5 indicate high, 3–3.99 moderate, 2–2.99 low, and ≤1.99 very low levels of digital fatigue/addiction.

#### 2.4.3. Depression, Anxiety, and Stress Scale (DASS-21)

The DASS-21 is a self-report instrument developed by Lovibond and Lovibond (1995) as an abbreviated version of the 42-item Depression, Anxiety, and Stress Scale (DASS-42) to assess symptoms of depression, anxiety, and stress [17]. The Turkish validity and reliability study was conducted by Sarıçam (2018) [13]. The scale comprises 21 items rated on a four-point Likert scale (0–3), with seven items for each of the three dimensions: depression, anxiety, and stress [13]. Scores of ≥5 on the depression subscale, ≥4 on the anxiety subscale, and ≥8 on the stress subscale indicate the presence of clinically relevant symptoms in the respective domain. To determine the severity levels, the raw scores obtained from the 7-item DASS-21 subscales were multiplied by two, as per the standard protocol, to align with the DASS-42 classification system. In the Turkish validation study, Cronbach’s alpha internal consistency coefficients were 0.87 for the depression subscale, 0.85 for the anxiety subscale, and 0.81 for the stress subscale.

### 2.5. Data Analysis

Statistical analyses were performed using IBM SPSS Statistics version 30.0 (IBM Corp., Armonk, NY, USA). Cronbach’s alpha coefficients were calculated to assess the internal consistency of the study instruments. Normality was evaluated by examining skewness and kurtosis coefficients as well as visual inspections of histograms, normal Q–Q plots, and boxplots. In line with Tabachnick and Fidell (2019), skewness and kurtosis values within ±1.5 were considered indicative of an approximately normal distribution [18]. Parametric tests were applied for variables meeting the normality assumption.

Descriptive statistics (frequency, percentage, mean, standard deviation, median, minimum, and maximum) were computed to summarize participants’ sociodemographic characteristics and scale scores. For comparisons between two independent groups (e.g., gender, smoking status), the independent-samples *t* test was used for normally distributed variables, whereas the Mann–Whitney U test was used for non-normally distributed variables. For comparisons across three or more groups (e.g., grade level, income status, daily internet use duration, sleep duration), one-way ANOVA was applied for normally distributed variables and the Kruskal–Wallis H test for non-normally distributed variables. When the assumption of homogeneity of variances was violated, Welch’s ANOVA was used. For ANOVA results indicating significant differences, appropriate post hoc tests were conducted according to variance homogeneity (Bonferroni or Games–Howell) to identify the source of the differences.

Pearson correlation analysis was conducted to examine associations among the total and subscale scores. Multivariable linear regression analyses were performed to identify which digital fatigue sub-dimensions were associated with students’ depression, anxiety, and stress levels. Statistical significance was determined based on *p*-values, with a threshold of *p* < 0.05. Due to the large number of group comparisons performed, results with *p*-values close to the 0.05 threshold were interpreted with caution to minimize the risk of Type I errors.

In this study, the Cronbach’s alpha internal consistency coefficients were found to be 0.936 for the DFS Total and 0.907 for the DASS-21 Total. For the DFS sub-dimensions, the alpha values were 0.922 for Digital Addiction, 0.869 for Online Pressure, 0.845 for Physical–Mental Fatigue, and 0.749 for Psychosomatic Problems. For the DASS-21 sub-dimensions, the alpha values were 0.842 for Depression, 0.751 for Anxiety, and 0.798 for Stress. These values indicate that both scales and their sub-dimensions have high reliability in the current sample.

The reporting of this study follows the Strengthening the Reporting of Observational Studies in Epidemiology (STROBE) guidelines. To ensure data integrity, only fully completed questionnaires were included in the final analysis, and there was no missing data among the 543 participants included in the study.

### 2.6. Ethical Consideration

The study was conducted in accordance with the Declaration of Helsinki and ethical approval to conduct the study was obtained from the relevant Ethics Committee of Ankara University (approval no.: 11/138; date: 16 June 2025), and institutional permission was granted by the Faculty of Nursing, Ankara University. Written informed consent was obtained from all students who agreed to participate. Permission to use the study instruments was obtained from the respective scale developers/owners.

## 3. Results

The sociodemographic characteristics of the participating students are presented in Table 1. The mean age of the students was 21.19 ± 1.55 years (range: 18–26), with 89.0% female and 32.0% being fourth-year students. It was observed that 71.5% of the participants resided in dormitories, 55.1% reported their income to be equal to their expenses, 84.5% were non-smokers, and 65.9% slept an average of 7 h or less per day. Regarding technology usage, 40.9% of the students reported using the internet for an average of 5–6 h daily, and 37.4% used the internet for educational purposes for an average of 1–2 h weekly. Additionally, 99.3% of the participants owned a phone, and 79.6% used digital devices primarily for media purposes.

Descriptive statistics for the scales and their sub-dimensions are presented in Table 2. The mean total score for the DASS-21 was 17.18 (SD = 9.49), ranging from 0 to 45. The mean total score for the DFS was 2.82 (SD = 0.68), with a range of 1 to 4.54.

The comparison of DASS-21 total and subscale scores according to students’ sociodemographic characteristics is presented in Table 3. Female students had significantly higher anxiety and stress scores compared to males (*p* = 0.042 and *p* = 0.029, respectively). In terms of grade level, third-year students exhibited significantly higher depression, stress, and DASS-21 total scores compared to second- and fourth-year students (all *p* ≤ 0.003). Students with income less than their expenses had significantly higher depression scores than those with income exceeding their expenses (*p* = 0.033). Those who used the internet for more than 6 h daily showed significantly higher depression, stress, and DASS-21 total scores, particularly compared to those using it 3–4 h per day (all *p* < 0.05). Students who slept 7 h or less per day had significantly higher stress scores than those who slept 8 h or more (*p* = 0.046). Moreover, students who smoked had significantly higher anxiety, stress, and DASS-21 total scores compared to non-smokers (all *p* ≤ 0.002).

The comparison of DFS total and sub-dimension scores according to students’ sociodemographic characteristics is presented in Table 4. Among age groups, a significant difference was found only in the online pressure scores, with the 22-year-old group scoring higher than the 20-year-old group (*p* = 0.005). Female students had significantly higher scores than males in digital addiction, physical–mental fatigue, and total DFS scores (*p* = 0.006, *p* < 0.001, and *p* = 0.005, respectively). Regarding grade level, fourth-year students had significantly higher online pressure scores compared to first- and second-year students (*p* < 0.05). As daily internet usage increased, scores for digital addiction, physical–mental fatigue, psychosomatic problems, and total DFS also increased significantly; notably, students using the internet for more than 6 h daily had higher scores in all these sub-dimensions and the total score compared to those using it for 1–2 and 3–4 h (*p* < 0.001 for all). These findings indicate that prolonged internet use is associated with both digital addiction and all components of digital fatigue.

The correlation coefficients between the DASS-21 and DFS scales are presented in Table 5. The total DASS-21 score showed a statistically significant positive correlation with the total DFS score (r = 0.458, *p* < 0.001) and the DFS psychosomatic problems subscale (r = 0.438, *p* < 0.001), both at a moderate level. Positive moderate correlations were also found with the DFS digital addiction (r = 0.397, *p* < 0.001) and DFS physical and mental fatigue (r = 0.376, *p* < 0.001) subscales. Additionally, a statistically significant positive but weak correlation was observed with the DFS online pressure subscale (r = 0.272, *p* < 0.001).

The results of the multivariable regression analysis examining the relationship between digital fatigue levels and depression, anxiety, and stress levels are presented in Table 6. Before conducting the regression analyses, collinearity diagnostics were performed. It was found that the Variance Inflation Factor (VIF) values for all independent variables were below 3.0 (ranging between 1.25 and 2.80), and Tolerance values were above 0.2, indicating the absence of multicollinearity issues in the models. The standardized coefficients (*β*) indicated that the associations were of moderate magnitude according to Cohen’s benchmarks. The consistency of these findings highlights that digital fatigue, particularly its psychosomatic manifestations, explains a substantial portion of the variance in mental health outcomes among nursing students.

Model 1 (Depression): The regression model was found to be significant and well-fitted (F (4, 538) = 25.938, *p* < 0.001), explaining 16.2% of the total variance (R^2^ = 0.162). The analysis revealed that significant predictors of depression levels were the DFS digital addiction subscale (β = 0.178, *p* = 0.001), online pressure (β = 0.104, *p* = 0.026), and psychosomatic problems (β = 0.174, *p* = 0.005). These findings indicate that all these predictors are positively associated with depression levels.

Model 2 (Anxiety): The regression model was significant and well-fitted (F (4, 538) = 28.679, *p* < 0.001), explaining 17.6% of the total variance (R^2^ = 0.176). The analysis identified the DFS psychosomatic problems subscale (β = 0.236, *p* < 0.001) and physical and mental fatigue (β = 0.156, *p* = 0.003) as significant predictors of anxiety levels. These results show that these predictors are positively associated with anxiety levels.

Model 3 (Stress): The regression model was significant and well-fitted (F (4, 538) = 33.923, *p* < 0.001), explaining 20.1% of the total variance (R^2^ = 0.201). The regression analysis revealed that significant predictors of stress levels were the DFS digital addiction subscale (β = 0.180, *p* = 0.001), DFS psychosomatic problems subscale (β = 0.210, *p* <0.001), and physical and mental fatigue (β = 0.157, *p* = 0.002). These findings indicate that all these predictors are positively associated with stress levels.

## 4. Discussion

This study evaluated the levels of depression, anxiety, stress, and digital fatigue among nursing students and examined the relationships between the sub-dimensions of digital fatigue and depression, anxiety, and stress. The findings indicate that depressive symptoms, anxiety, and stress are present at significant rates among nursing students, and digital fatigue levels are notably elevated. In particular, the increase in daily internet usage was associated with higher scores of depression, stress, and digital fatigue. Additionally, female students exhibited higher levels of anxiety, stress, and digital fatigue, highlighting the importance of risk factors related to both digital lifestyle and gender. However, as these specific findings (e.g., gender differences in anxiety, *p* = 0.042) are close to the significance threshold, they should be interpreted conservatively within the context of multiple testing. While this study primarily focused on the predictive power of digital fatigue sub-dimensions, it is important to acknowledge the role of potential confounding variables. Our univariate analyses indicated that factors such as gender, sleep quality, and daily internet duration are significantly associated with psychological distress. These sociodemographic factors likely interact with digital fatigue, suggesting that digital health interventions should be tailored to the specific needs of vulnerable groups, particularly those with high digital exposure and poor sleep hygiene. Our finding that digital fatigue levels are notably elevated among nursing students aligns with global trends. In comparison with international literature, our results are consistent with Romero-Rodríguez et al. (2023), who identified digital fatigue as a significant consequence of online learning in Spain, and Emmamally et al. (2024), who described similar digital exhaustion specifically among nursing students in South Africa [1,3]. These cross-cultural similarities confirm that digital fatigue is an emerging global challenge within nursing education.

Our finding of a high prevalence of depression and anxiety among nursing students is consistent with the existing literature. Tung et al. (2018) reported that symptoms of depression and anxiety are common among nursing students and may negatively impact academic performance and clinical competence [15]. Another study found that anxiety and stress levels significantly increase during clinical practicum periods among nursing students [19]. Research indicates that rates of depression and anxiety in nursing students are substantial, suggesting that this psychological burden may be related to the intensity of the educational process and clinical responsibilities [13,19]. These findings further highlight the psychosocial impacts of nursing education.

In our study, female students exhibited higher levels of anxiety, stress, and digital fatigue—particularly digital addiction and physical–mental fatigue—compared to male students, which aligns with the literature. Tung et al. (2018) identified female gender as a potential risk factor for depression and anxiety among nursing students [15]. Other studies on university students have also shown that females tend to have higher depression and anxiety scores and are more likely to express psychological symptoms [20]. These differences may be related to additional responsibilities imposed by gender roles, differences in help-seeking behaviors, and cultural expectations regarding emotional expression. Regarding digital use, some studies suggest that female students’ more intensive use of social media and communication-focused applications may increase online pressure and social comparison, thereby exacerbating psychological burden [5].

The observed increase in depression, stress, and digital fatigue levels with longer daily internet use aligns with the extensive literature demonstrating the relationship between problematic digital use and psychological symptoms among university students. Demirci et al. (2015) showed that the intensity of smartphone use is associated with impaired sleep quality and increased depression and anxiety scores [4]. Elhai et al. (2017) found that problematic smartphone use is systematically linked to anxiety and depression psychopathology [5]. Alimoradi et al. (2019) reported a strong association between internet addiction and sleep disturbances, noting that poor sleep quality may exacerbate depressive symptoms [6]. A meta-analysis revealed that problematic internet use is moderately associated with depression and anxiety, particularly in adolescents and young adults [21]. A study conducted among adolescents reported that individuals who use the internet for more than three hours per day have higher levels of psychosocial problems [22]. Consistent with this evidence, our study found that students using the internet for more than 6 h daily had significantly higher levels of depression, stress, and digital fatigue, supporting the potential impact of digital exposure on both psychological symptoms and perceived fatigue.

When examining the sub-dimensions of the Digital Fatigue Scale, our study found that digital addiction, physical and mental fatigue, and psychosomatic problems were positively and significantly correlated with depression, anxiety, and stress. Moreover, these sub-dimensions emerged as significant predictors of depression, anxiety, and stress in multivariable regression models. This finding suggests that digital fatigue is not merely a subjective feeling of “tiredness” but is associated with a broader psychological stress profile involving cognitive overload, concentration difficulties, sleep disturbances, and somatic complaints [11,12]. Other studies have also demonstrated that intensive use of digital devices is linked to symptoms such as eye and headache pain, neck and back pain, chronic fatigue, and sleep disorders, which can co-occur with depressive mood and anxiety symptoms [4,6,23]. In particular, online pressure and constant exposure to notifications can increase anxiety and restlessness through mechanisms such as Fear of Missing Out (FOMO) and a state of hyperarousal [5,24]. Significantly, our multivariable regression models revealed that online pressure and digital addiction are critical drivers of depression, while psychosomatic problems emerged as a particularly strong predictor for both anxiety and stress. The consistent significance of Psychosomatic Problems across all models may partially stem from shared item content with the DASS-21 (e.g., physical exhaustion). However, the DFS specifically frames these symptoms within the context of digital device use, distinguishing them from generalized somatic distress. This finding highlights the clinical interplay between digital usage patterns and mental health. The strong predictive power of psychosomatic problems suggests that the psychological burden of digital fatigue manifests through physical symptoms such as headaches and eye strain, creating a feedback loop that exacerbates students’ stress levels. Linking these digital components directly to psychological distress underscores that digital fatigue is not merely a technical issue but a complex health phenomenon requiring physical and mental interventions.

These findings indicate that the digital burden encountered by nursing students during their education has significant implications not only for academic performance but also for mental health and physical well-being. Nursing education in Türkiye is conducted through a hybrid curriculum comprising intensive theoretical courses, clinical practice, and an increasing number of digital components, which leads to continuous exposure to digital devices among students [13,14]. The widespread adoption of online and hybrid learning models during the COVID-19 pandemic has been highlighted in numerous studies as further increasing screen time and the risk of digital exhaustion [25,26]. Although this study was conducted post-pandemic, it is likely that remote and hybrid education experiences have permanently altered students’ digital habits. Therefore, it is important that nursing education programs incorporate structured training and counseling aimed at enhancing digital literacy and digital health concepts, as well as managing screen time, promoting sleep hygiene, coping with stress, and strengthening self-care skills.

The findings of our study suggest that regular mental health screenings and assessments of digital fatigue levels could be beneficial for nursing students. Monitoring depression, anxiety, and stress levels using brief and easy-to-administer scales such as the DASS-21 can facilitate the identification of high-risk students and enable timely referrals to psychological counseling, peer support groups, and psychiatric evaluation when necessary [13]. For students at high risk of digital fatigue, interventions such as structured screen time management, digital detox programs, relaxation exercises, and physical activity promotion can be planned. At the institutional level, policies aimed at optimizing the timing of online assignments and announcements, reducing notification overload, and alleviating the pressure on students to remain constantly online may contribute to reducing digital fatigue.

## 5. Strengths and Limitations

This study has several strengths. The relatively large sample size and inclusion of all class levels from a single nursing faculty enhance the representativeness of the findings within the context of the institution. Examining the relationships between digital fatigue and depression, anxiety, and stress not only at a correlational level but also through multivariable regression analyses allowed for a more detailed assessment of the contribution of digital fatigue components to psychological symptoms. However, some limitations should be considered. Due to the cross-sectional design, the relationships between digital fatigue and depression, anxiety, and stress cannot be interpreted causally; only concurrent associations can be identified. The use of self-report scales for data collection may introduce biases such as social desirability and recall bias.

The cross-sectional design of this study precludes the establishment of causal relationships. The observed associations between digital fatigue and mental health outcomes may be bidirectional; for instance, students with pre-existing anxiety may be more prone to experiencing digital fatigue. Future longitudinal research is needed to determine the temporal sequence of these variables. As the study was conducted in a single nursing faculty in Türkiye, the findings may not be fully generalizable to students in different cultural or educational systems, despite the global nature of nursing education requirements.

The response rate was 65%, and it is possible that non-respondents differed in their digital habits or mental health status, potentially leading to a participation bias.

The study was conducted in a single university’s nursing faculty, which limits the generalizability of the findings to different institutional and cultural contexts. Additionally, since structured clinical interviews were not conducted, depression, anxiety, and stress levels were assessed solely based on self-reported symptoms.

While this study identifies digital fatigue as a core correlate of psychological distress, it is important to acknowledge the potential for reverse causality; students with pre-existing depression or anxiety may shift toward increased digital media consumption as a coping mechanism, thereby exacerbating digital exhaustion.

## 6. Conclusions

This study revealed a strong and significant relationship between digital fatigue and levels of depression, anxiety, and stress among nursing students. The findings indicate that female gender and daily internet use of six hours or more are important risk factors associated with higher scores in both psychological symptoms and digital fatigue. Specifically, components of digital fatigue such as digital addiction, physical–mental fatigue, and psychosomatic problems were found to significantly predict students’ levels of depression, anxiety, and stress.

Based on these results, it is recommended to integrate digital health, screen time management, and digital balance strategies into the nursing education curriculum; to regularly screen students’ mental health within the faculty; and to develop psychosocial support programs targeting at-risk groups. Implementing institutional policies to reduce digital fatigue will support the academic success and professional well-being of future nurses. Future research examining the long-term effects of digital fatigue on clinical performance and professional burnout will provide valuable contributions to the literature.

Future studies should explore the effectiveness of interventions aimed at reducing digital fatigue and promoting mental well-being among nursing students. Qualitative and mixed-method research is recommended to examine how screen-time management, sleep hygiene, and stress-coping education impact students’ psychological resilience and academic performance. Additionally, research should investigate the role of institutional strategies, such as optimized online workload and notification management, in mitigating digital overload. Longitudinal studies could provide insight into how digital fatigue affects students’ professional development, risk of burnout, and readiness for clinical practice, ultimately supporting safe patient care.

Policymakers in higher education and health ministries should develop national guidelines for ‘digital ergonomics’ in health sciences curricula. We suggest implementing ‘digital-free zones’ in campuses and regulating the volume of mandatory online assignments to prevent digital burnout among future healthcare professionals.

## Figures and Tables

**Table 1:** Distribution of students’ sociodemographic characteristics (N = 543).

Characteristics	Mean ± SD/n	Min.–Max./%
**Age (years)**	21.19 ± 1.55	18–26
**Age Group**		
18	23	4.2
19	52	9.6
20	109	20.1
21	121	22.3
22	134	24.7
23	71	13.1
24 years and above	33	6.1
**Gender**		
Female	483	89.0
Male	60	11.0
**Grade**		
1st year	84	15.5
2nd year	133	24.5
3rd year	152	28.0
4th year	174	32.0
**Income Status**		
My income is less than my expenses	179	33.0
My income is equal to my expenses	299	55.1
My income is more than my expenses	65	12.0
**Average Daily Internet Use**		
1–2 h	25	4.6
3–4 h	212	39.0
5–6 h	222	40.9
More than 6 h	84	15.5
Owned Technological Devices *		
Phone	539	99.3
Computer	268	49.4
Tablet	269	49.5
Purpose of Using Digital Devices *		
Education	216	39.8
Media	432	79.6
Communication	262	48.3
Gaming	108	19.9
Music/Video	355	65.4
Internet Browsing	174	32.0
**Average Weekly Internet Use for Education**		
Less than 1 h	117	21.5
1–2 h	203	37.4
3–4 h	133	24.5
5–6 h	60	11.0
More than 6 h	30	5.5
**Average Daily Sleep Duration**		
7 h or less	358	65.9
8 h or more	185	34.1
**Place of Residence**		
Dormitory	388	71.5
At home with friends	26	4.8
At home with family	129	23.8
**Smoking Status**		
Yes	84	15.5
No	459	84.5

* Multiple responses were allowed (percentage values calculated based on n = 543).

**Table 2:** Descriptive statistics for scales and sub-dimensions.

		Observed Range	Expected Range
**Scales and Sub-dimensions**	Mean ± SD	Min.–Max.	Min.–Max.
**DASS-21 Total**	17.18 ± 9.49	0–45	0–63
Depression	5.76 ± 3.88	0–20	0–21
Anxiety	4.88 ± 3.29	0–15	0–21
Stress	6.54 ± 3.65	0–18	0–21
**DFS Total**	2.82 ± 0.68	1–4.54	1–5
Digital Addiction	3.12 ± 0.83	1–5	1–5
Online Pressure	2.19 ± 0.78	1–4.43	1–5
Physical–Mental Fatigue	3.06 ± 0.90	1–5	1–5
Psychosomatic Problems	2.72 ± 0.88	1–5	1–5

**Table 3:** Comparison of mean total and subscale DASS-21 scores according to students’ sociodemographic characteristics (N = 543).

Characteristics	n	Depression Mean ± SD	Anxiety Mean ± SD	Stress Mean ± SD	DASS-21 Total Mean ± SD
**Age Groups**					
18	23	6.39 ± 4.61	4.74 ± 3.39	7.96 ± 4.79	19.09 ± 10.85
19	52	6.15 ± 4.29	5.19 ± 3.22	6.56 ± 3.38	17.90 ± 9.61
20	109	5.44 ± 3.70	4.50 ± 3.07	6.36 ± 3.91	16.29 ± 9.03
21	121	6.30 ± 3.89	5.26 ± 3.46	6.93 ± 3.58	18.49 ± 9.55
22	134	5.58 ± 3.81	5.15 ± 3.39	6.49 ± 3.51	17.22 ± 9.71
23	71	5.76 ± 3.65	4.41 ± 3.12	6.11 ± 3.35	16.28 ± 8.78
24+	33	4.52 ± 3.93	4.33 ± 3.31	5.79 ± 3.66	14.64 ± 9.94
**Test**		χ^2^ = 8.004	F = 1.143	F = 1.260	F = 1.251
***p***		0.238	0.336	0.274	0.279
**Gender**					
Female	483	5.72 ± 3.84	4.98 ± 3.30	6.66 ± 3.66	17.36 ± 9.48
Male	60	6.10 ± 4.21	4.07 ± 3.16	5.57 ± 3.50	15.73 ± 9.52
**Test**		t = −0.717	**t = 2.041**	**t = 2.191**	t = 1.253
***p***		0.473	**0.042**	**0.029**	0.211
**Grade**					
1st year ^1^	84	6.11 ± 4.24	4.81 ± 3.22	7.01 ± 3.80	17.93 ± 9.67
2nd year ^2^	133	5.28 ± 3.59	4.66 ± 3.12	6.00 ± 3.41	15.94 ± 8.35
3rd year ^3^	152	6.92 ± 4.03	5.49 ± 3.57	7.42 ± 3.96	19.83 ± 10.41
4th year ^4^	174	4.95 ± 3.54	4.56 ± 3.16	5.95 ± 3.31	15.45 ± 8.88
**Test**		**χ^2^ = 22.281**	F = 2.509	**W = 5.734**	**F = 7.021**
***p***		**<0.001**	0.058	**0.001**	**<0.001**
**Differences**		**3 > 4; *p* < 0.001**		**3 > 4; *p* = 0.002**	**3 > 4; *p* < 0.001**
		**3 > 2; *p* = 0.003**		**3 > 2; *p* = 0.007**	**3 > 2; *p* = 0.003**
**Income Status**					
Income less than expenses ^1^	179	6.32 ± 4.06	5.24 ± 3.14	6.81 ± 3.69	18.37 ± 9.51
Income equal to expenses ^2^	299	5.62 ± 3.84	4.72 ± 3.36	6.42 ± 3.62	16.76 ± 9.48
Income more than expenses ^3^	65	4.89 ± 3.38	4.66 ± 3.33	6.31 ± 3.70	15.86 ± 9.32
**Test**		**F = 3.717**	F = 1.592	F = 0.769	F = 2.341
***p***		**0.025**	0.204	0.464	0.097
**Differences**		**1 > 3; *p* = 0.033**			
**Average Daily Internet Use**					
1–2 h ^1^	25	4.92 ± 3.55	4.48 ± 2.68	5.56 ± 3.08	14.96 ± 7.90
3–4 h ^2^	212	5.31 ± 3.62	4.62 ± 3.35	6.08 ± 3.54	16.00 ± 9.35
5–6 h ^3^	222	5.81 ± 3.80	4.97 ± 3.28	6.67 ± 3.43	17.45 ± 9.12
More than 6 h ^4^	84	7.04 ± 4.56	5.42 ± 3.29	7.65 ± 4.36	20.11 ± 10.61
**Test**		**F = 4.469**	F = 1.362	**W = 3.858**	**F = 4.339**
***p***		**0.004**	0.254	**0.012**	**0.005**
**Differences**		**4 > 2; *p* = 0.003**		**4 > 2; *p* = 0.019**	**4 > 2; *p* = 0.005**
				**4 > 1; *p* = 0.045**	
**Average Daily Sleep Duration**					
7 h or less	358	5.83 ± 3.90	4.90 ± 3.24	6.76 ± 3.74	17.49 ± 9.55
8 h or more	185	5.62 ± 3.85	4.85 ± 3.40	6.10 ± 3.44	16.57 ± 9.37
**Test**		t = 0.599	t = 0.170	**t = 2.000**	t = 1.072
***p***		0.549	0.865	**0.046**	0.284
**Place of Residence**					
Dormitory	388	5.74 ± 3.76	4.85 ± 3.19	6.55 ± 3.55	17.14 ± 9.15
At home with friends	26	6.73 ± 3.84	6.04 ± 3.93	6.88 ± 3.94	19.65 ± 10.31
At home with family	129	5.63 ± 4.25	4.74 ± 3.43	6.43 ± 3.92	16.80 ± 10.31
**Test**		F = 0.892	F = 1.741	F = 0.179	F = 0.990
***p***		0.411	0.176	0.836	0.372
**Smoking Status**					
Yes	84	6.44 ± 4.10	6.06 ± 3.43	7.69 ± 3.96	20.19 ± 10.21
No	459	5.64 ± 3.84	4.67 ± 3.22	6.33 ± 3.56	16.63 ± 9.26
**Test**		t = 1.748	**t = 3.606**	**t = 3.172**	**t = 3.188**
***p***		0.081	**<0.001**	**0.002**	**0.002**

t: Independent samples *t*-test value, F: One-way analysis of variance test value (ANOVA), W: Welch test value, χ^2^: Kruskal–Wallis H test value, 1-2-3-4: Indicators of differences between groups. Bold values indicate statistical significance (*p* < 0.05).

**Table 4:** Comparison of mean DFS total and sub-dimension scores according to students’ sociodemographic characteristics (N = 543).

Characteristic	n	Digital Addiction Mean ± SD	Online Pressure Mean ± SD	Physical–Mental Fatigue Mean ± SD	Psychosomatic Problems Mean ± SD	DFS Total Mean ± SD
**Age Groups**						
18 ^1^	23	3.13 ± 0.80	1.94 ± 0.87	3.02 ± 0.80	2.57 ± 0.71	2.73 ± 0.58
19 ^2^	52	3.08 ± 0.86	2.11 ± 0.77	3.04 ± 0.82	2.71 ± 0.96	2.78 ± 0.66
20 ^3^	109	3.12 ± 0.91	2.01 ± 0.70	3.02 ± 0.94	2.58 ± 0.94	2.75 ± 0.71
21 ^4^	121	3.16 ± 0.78	2.24 ± 0.81	3.08 ± 0.91	2.80 ± 0.89	2.86 ± 0.68
22 ^5^	134	3.21 ± 0.77	2.29 ± 0.79	3.17 ± 0.90	2.82 ± 0.83	2.91 ± 0.65
23 ^6^	71	2.97 ± 0.79	2.29 ± 0.81	2.99 ± 0.88	2.60 ± 0.74	2.75 ± 0.63
24+ ^7^	33	2.97 ± 1.01	2.29 ± 0.71	2.99 ± 1.04	2.91 ± 1.06	2.79 ± 0.83
**Test**		F = 0.870	**F = 2.163**	F = 0.502	F = 1.582	F = 0.977
***p***		0.516	**0.045**	0.807	0.150	0.440
**Difference**			**5 > 3; *p* = 0.005**			
**Gender**						
Female	483	3.15 ± 0.81	2.19 ± 0.77	3.13 ± 0.88	2.74 ± 0.88	2.85 ± 0.66
Male	60	2.84 ± 0.92	2.21 ± 0.86	2.53 ± 0.91	2.55 ± 0.86	2.59 ± 0.76
**Test**		**t = 2.751**	t = −0.258	**t = 4.973**	t = 1.653	**t = 2.847**
***p***		**0.006**	0.797	**<0.001**	0.099	**0.005**
**Grade**						
1st year ^1^	84	3.17 ± 0.82	2.03 ± 0.74	2.91 ± 0.84	2.59 ± 0.87	2.76 ± 0.63
2nd year ^2^	133	3.07 ± 0.86	2.05 ± 0.78	3.03 ± 0.95	2.64 ± 0.92	2.75 ± 0.71
3rd year ^3^	152	3.16 ± 0.81	2.24 ± 0.78	3.13 ± 0.91	2.82 ± 0.86	2.87 ± 0.66
4th year ^4^	174	3.09 ± 0.83	2.33 ± 0.78	3.11 ± 0.88	2.77 ± 0.87	2.86 ± 0.69
**Test**		F = 0.451	**F = 4.633**	F = 1.259	F = 1.817	F = 1.273
***p***		0.717	**0.003**	0.288	0.143	0.283
**Difference**			**4 > 2; *p* = 0.013**			
			**4 > 1; *p* = 0.022**			
**Income Status**						
Income less than expenses	179	3.11 ± 0.81	2.21 ± 0.75	3.14 ± 0.89	2.74 ± 0.88	2.84 ± 0.65
Income equal to expenses	299	3.12 ± 0.84	2.19 ± 0.79	3.05 ± 0.86	2.73 ± 0.87	2.82 ± 0.68
Income more than expenses	65	3.12 ± 0.87	2.13 ± 0.83	2.92 ± 1.08	2.63 ± 0.97	2.77 ± 0.76
**Test**		F = 0.010	F = 0.235	W = 1.173	F = 0.405	F = 0.265
***p***		0.990	0.791	0.312	0.667	0.768
**Average Daily Internet Use**						
1–2 h ^1^	25	2.63 ± 0.80	2.08 ± 0.69	2.71 ± 0.93	2.19 ± 0.78	2.44 ± 0.64
3–4 h ^2^	212	2.98 ± 0.77	2.13 ± 0.76	2.91 ± 0.88	2.58 ± 0.85	2.70 ± 0.64
5–6 h ^3^	222	3.18 ± 0.80	2.23 ± 0.80	3.15 ± 0.89	2.79 ± 0.86	2.88 ± 0.67
More than 6 h ^4^	84	3.46 ± 0.91	2.27 ± 0.82	3.32 ± 0.89	3.07 ± 0.88	3.08 ± 0.70
**Test**		**F = 10.699**	F = 1.011	**F = 6.499**	**F = 10.160**	**F = 10.202**
***p***		**<0.001**	0.388	**<0.001**	**<0.001**	**<0.001**
**Difference**		**4 > 2; *p* < 0.001**		**4 > 2; *p* = 0.002**	**4 > 1; *p* < 0.001**	**4 > 2; *p* < 0.001**
		**4 > 1; *p* < 0.001**		**4 > 1; *p* = 0.016**	**4 > 2; *p* < 0.001**	**4 > 1; *p* < 0.001**
		**3 > 1; *p* = 0.009**		**3 > 2; *p* = 0.028**	**3 > 1; *p* = 0.006**	**3 > 1; *p* = 0.011**
		**4 > 3; *p* = 0.034**				**3 > 2; *p* = 0.024**
**Average Daily Sleep Duration**						
7 h or less	358	3.10 ± 0.85	2.16 ± 0.78	3.05 ± 0.90	2.73 ± 0.87	2.80 ± 0.68
8 h or more	185	3.15 ± 0.79	2.25 ± 0.78	3.08 ± 0.91	2.71 ± 0.91	2.85 ± 0.66
**Test**		t = −0.645	t = −1.362	t = −0.362	t = 0.254	t = −0.771
***p***		0.519	0.174	0.718	0.799	0.441
**Place of Residence**						
Dormitory	388	3.12 ± 0.80	2.19 ± 0.79	3.06 ± 0.89	2.72 ± 0.89	2.82 ± 0.67
At home with friends	26	3.13 ± 0.96	2.35 ± 0.93	3.23 ± 1.04	2.84 ± 1.07	2.91 ± 0.83
At home with family	129	3.09 ± 0.91	2.14 ± 0.74	3.04 ± 0.90	2.72 ± 0.82	2.79 ± 0.67
**Test**		W = 0.061	F = 0.782	F = 0.494	W = 0.158	F = 0.347
***p***		0.941	0.458	0.610	0.854	0.707
**Smoking Status**						
Yes	84	3.11 ± 0.79	2.11 ± 0.80	3.18 ± 1.04	2.84 ± 0.89	2.84 ± 0.67
No	459	3.12 ± 0.84	2.20 ± 0.78	3.04 ± 0.87	2.70 ± 0.88	2.82 ± 0.68
**Test**		t = −0.071	t = −0.965	t = 1.094	t = 1.353	t = 0.228
***p***		0.943	0.335	0.277	0.177	0.820

t: Independent samples *t*-test value, F: One-way analysis of variance test value (ANOVA), W: Welch test value, 1-2-3-4-5-6-7: Indicators of differences between groups. Bold values indicate statistical significance.

**Table 5:** Correlation coefficients between DASS-21 and DFS scales and their sub-dimensions (N = 543).

	Variables	1	2	3	4	5	6	7	8	9
1	DASS-21 Total	1								
2	DASS Depression	0.884 **	1							
		*p* < 0.001								
3	DASS Anxiety	0.865 **	0.647 **	1						
		*p* < 0.001	*p* < 0.001							
4	DASS Stress	0.879 **	0.652 **	0.657 **	1					
		*p* < 0.001	*p* < 0.001	*p* < 0.001						
5	DFS Total	0.458 **	0.396 **	0.391 **	0.418 **	1				
		*p* < 0.001	*p* < 0.001	*p* < 0.001	*p* < 0.001					
6	DFS Digital Addiction	0.397 **	0.349 **	0.309 **	0.382 **	0.896 **	1			
		*p* < 0.001	*p* < 0.001	*p* < 0.001	*p* < 0.001	*p* < 0.001				
7	DFS Online Pressure	0.272 **	0.278 **	0.250 **	0.187 **	0.683 **	0.409 **	1		
		*p* < 0.001	*p* < 0.001	*p* < 0.001	*p* < 0.001	*p* < 0.001	*p* < 0.001			
8	DFS Physical and Mental Fatigue	0.376 **	0.273 **	0.350 **	0.372 **	0.746 **	0.555 **	0.343 **	1	
		*p* < 0.001	*p* < 0.001	*p* < 0.001	*p* < 0.001	*p* < 0.001	*p* < 0.001	*p* < 0.001		
9	DFS Psychosomatic Problems	0.438 **	0.362 **	0.394 **	0.399 **	0.828 **	0.647 **	0.526 **	0.630 **	1
		*p* < 0.001	*p* < 0.001	*p* < 0.001	*p* < 0.001	*p* < 0.001	*p* < 0.001	*p* < 0.001	*p* < 0.001	

** Statistically significant results at the *p* < 0.001 level.

**Table 6:** Relationship between students’ digital fatigue levels and depression, anxiety, and stress levels: multivariable regression analysis.

	Dependent Variables								
Models	Model 1			Model 2			Model 3		
Independent Variables	Depression			Anxiety			Stress		
	β	t	*p*	β	t	*p*	β	t	*p*
Constant		−0.647	0.518		−0.619	0.536		0.436	0.663
DFS Digital Addiction	0.178	3.311	**0.001**	0.048	0.901	0.368	0.180	3.424	**0.001**
DFS Online Pressure	0.104	2.226	**0.026**	0.052	1.134	0.257	−0.051	−1.110	0.268
DFS Physical and Mental Fatigue	0.029	0.554	0.580	0.156	3.005	**0.003**	0.157	3.060	**0.002**
DFS Psychosomatic Problems	0.174	2.843	**0.005**	0.236	3.903	**<0.001**	0.210	3.532	**<0.001**
Model Summaries	F(4, 538) = 25.938, *p* < 0.001			F(4, 538) = 28.679, *p* < 0.001			F(4, 538) = 33.923, *p* < 0.001		
	R = 0.402; R^2^ = 0.162; Adj R^2^ = 0.155			R = 0.419; R^2^ = 0.176; Adj R^2^ = 0.170			R = 0.449; R^2^ = 0.201; Adj R^2^ = 0.195		
	T = 0.418; VIF = 2.394			T = 0.418; VIF = 2.394			T = 0.418; VIF = 2.394		
	Durbin Watson = 1.950			Durbin Watson = 2.059			Durbin Watson = 1.962		

β: Standardized regression coefficient; R^2^: Explained variance; Adj R^2^: Adjusted explained variance; T: Tolerance value; VIF: Variance inflation factor. Bold values indicate statistical significance.

## Data Availability

The data that support the findings of this study are not publicly available due to ethical and privacy restrictions but are available from the corresponding author upon reasonable request.

## Supplementary Materials

The following supporting information can be downloaded at: https://www.mdpi.com/article/10.3390/healthcare14131950/s1, File S1: The STROBE reporting checklist. References [27,28] are included in the Supplementary Materials.
