# Supplementary material for: Stress, Anxiety, Depression, and Digital Fatigue Among Nursing Students: A Cross-Sectional Study
Source: Healthcare (Basel). 2026 Jul 1;14(13):1950. doi: 10.3390/healthcare14131950 (PMC13361551; doi:10.3390/healthcare14131950)
Supplement: Supplementary file 1 [file healthcare-14-01950-s001.zip › healthcare-4403369-supplementary.pdf]

File S1: The STROBE reporting checklist

For checking that observational epidemiology research articles can be understood and used by everyone

How to use this reporting checklist

This reporting checklist allows authors to demonstrate that their manuscripts adhere to the STROBE reporting guideline.

If you have not used a reporting guideline before, read about how and why to use them and check whether STROBE is the most applicable reporting guideline for your work.

Reporting guidelines are most useful when used early in research. When writing a manuscript or application, consider using the full guidance where you'll find explanations and examples for each item.

After writing, demonstrate adherence by completing this checklist:

1. Specify where each item is described (see Note 1).
2. Cite this checklist (See Note 2).
3. Include your completed checklist as a supplement when submitting to a journal so that future readers can use it to find information.

	Item Description	Location (or reason for not reporting)
**Title and abstract**		
1a. Indicate the study's design	Title, Page 1; Abstract, Page 2	Design, page 6
1b. Abstract	Abstract, Page 2	Abstract, page 1
**Introduction**		
2. Background / rationale	Introduction, Pages 2-4	Introduction, page 3-5
3. Objectives	Introduction, Page 4 (Line 121-123); Research hypotheses, Page 4	Research hypotheses, page 5
**Methods**		
4. Study design	Methods, Section 2.1, Page 4	Design, page 6
5. Setting	Methods, Section 2.2, Page 4; Data Collection, Section 2.3, Page 5	Method, page 6-10
6a. Eligibility criteria	Methods, Section 2.3, Page 5 (Lines 167-172)	Method, page 6-10
6b. Matching criteria	Not applicable – cross-sectional study.	Not applicable – this is a cross-sectional study; no matching was performed

7. Variables	Methods, Section 2.4, Page 5-6 (Outcomes: DASS-21, DFS; Predictors: Digital fatigue sub-dimensions)	Outcome variables included stress, anxiety, depression, and digital fatigue, measured using standardized questionnaires. Exposure and predictor variables included age, gender, and academic year. Potential confounders such as sleep duration and daily digital device use were recorded. Standard cut-off points for each scale were used where applicable.
8. Data sources / measurement	Methods, Section 2.4, Page 5-6; Data collection instruments	Data collection instruments, Page 7-8<br>Measurement methods were identical across all participants.
9. Bias	Methods, Section 2.5, Page 6; Discussion, Section 5, Page 18	Efforts to minimize potential bias included using standardized and validated questionnaires, ensuring anonymity of responses, and applying consistent data collection procedures across all participants
10. Study size	Methods, Section 2.2, Page 4 (Formula provided)	Participants and sample size, Page 6-7
11. Quantitative variables	Methods, Section 2.5, Page 6	Data analysis, Page 9
12a. Statistical methods	Methods, Section 2.5, Page 6-7	Data analysis, Page 9
12b. Statistical methods – subgroups and interactions	Methods, Section 2.5, Page 6 (ANOVA, Kruskal-Wallis for sociodemographic comparisons)	Data analysis, Page 9
12c. Statistical methods – missing data	Methods, Section 2.5, Page 7 (Line 251: 'no missing data among the 543 participants')	There were no missing data in this study.
12di. Statistical methods – loss to follow-up	Not applicable – cross-sectional study.	Not applicable – this is a cross-sectional study; no matching of cases and controls was performed.
12dii. Statistical methods –	Not applicable – cross-sectional study.	Not applicable – this is a cross-sectional study; no matching

matching cases and controls		of cases and controls was performed.
12diii. Statistical methods – sampling strategy	Not applicable – cross-sectional study.	Not applicable – this is a cross-sectional study; no matching of cases and controls was performed.
12e. Statistical methods – sensitivity analyses	Not applicable – no sensitivity analyses were conducted.	Not applicable – no sensitivity analyses were conducted in this study.
**Results**		
13a. Participant numbers	Methods, Section 2.3, Page 5 (838 eligible, 543 completed)	Participants and sample size, Page 6<br>Data Collection, Page 7
13b. Participants – non-participation	Methods, Section 2.3, Page 5 (Response rate: ~65%)	A total of 543 students were included in the study. The remaining students did not participate as they declined to take part.
13c. Participants – flow diagram	Not included – participant flow described in the text.	Not included – participant flow was described in the text.
14a. Descriptive data – participant characteristics	Results, Section 3, Page 7-13; Table 1 (Page 7)	Participants' sociodemographic characteristics, exposure variables, and potential confounders are summarized in Table 1,
14b. Descriptive data – missing data	Methods, Section 2.5, Page 7 (No missing data)	There were no missing data for any variables in this study
14c. Descriptive data – follow-up time	Not applicable – cross-sectional study.	Not applicable – this is a cross-sectional study; no follow-up was conducted.
15. Outcome data	Results, Section 3, Page 8; Table 2 (Page 8)	Tables 2, 3, 4, 5, 6
16a. Main results	Results, Section 3, Page 15; Table 6 (Page 15) - Standardized beta and p-values.	Unadjusted and multivariable regression estimates with 95% confidence intervals for the associations between digital fatigue sub-dimensions and depression, anxiety, and stress levels are reported in Table 6. Sociodemographic variables were included as potential confounders based on previous literature.

16b. Main results – category boundaries	Results, Section 3; Table 1 (Page 7)	Continuous variables that were categorized (e.g., age, daily digital device use) and their category boundaries are presented in Table 1, page 22.
16c. Main results – risk	Not applicable – regression coefficients reported.	Not applicable – relative risk estimates are not relevant for this cross-sectional study.
17. Other analyses	Results, Section 3, Page 9-13 (Comparison by sociodemographic variables); Tables 3-5	No additional analyses such as subgroup or sensitivity analyses were conducted in this study.
**Discussion**		
18. Key results	Discussion, Section 4, Page 16 (Paragraph 1)	-Nursing students reported high levels of depression, anxiety, and stress, along with moderate digital fatigue.<br>-Digital fatigue and psychosomatic/physical–mental issues were significant predictors of psychological symptoms, particularly among female students and those using the internet ≥6hours/day.<br>-Integrating digital well-being and screen-time management into nursing education programs may help support students' mental health.
19. Limitations	Discussion, Section 5, Page 18	Limitations, Page 16
20. Interpretation	Discussion, Section 4, Page 16-18	Recommendations and future research, Page 17
21. Generalisability	Discussion, Section 5, Page 18	he findings of this study may have limited generalisability because the sample was drawn from a single nursing department at one university. Differences in institutional policies, cultural context, and educational programs may affect the applicability of the results to other nursing student populations
**Other information**		

22. Funding	Other information, Page 19 (Line 533)	This study received no specific funding from public, commercial, or not-for-profit sectors.

1 How to specify where content is

Tell the reader where they can find information. E.g.,

- Results; paragraph 2
- Methods, Participants; paragraphs 1 & 2.
- Table 3
- Supplement B, para. 4

If you have chosen not to describe an item, explain why. You can do this in the checklist, or as a note below it.

You can describe items in the article body, or in tables, figures, or supplementary materials, and should prioritize items you feel are most important to your intended audience. The order of items in your manuscript does not need to match the order of items in this checklist. You can decide how best to structure your work.

2 How to cite

Describe how you used STROBE at the end of your Methods section, referencing the resources you used e.g.,

> 'We used the STROBE reporting guideline(1) to draft this manuscript, and the STROBE reporting checklist(2) when editing, included in supplement A'

If you use a reporting checklist, remember to include it as a supplement when publishing so that readers can easily find information and see how you have interpreted the guidance.

1. Elm E von, Altman DG, Egger M, Pocock SJ, Gøtzsche PC, Vandenbroucke JP, et al. The strengthening the reporting of observational studies in epidemiology (STROBE) statement: Guidelines for reporting observational studies. Annals of Internal Medicine [Internet]. 2007 Oct;147(8):573–7. Available from: https://www.acpjournals.org/doi/10.7326/0003-4819-147-8-200710160-00010

2. Elm E von, Altman DG, Egger M, Pocock SJ, Gøtzsche PC, Vandenbroucke JP, et al. The STROBE reporting checklist. In: Harwood J, Albury C, Beyer J de, Schlüssel M, Collins G, editors. The EQUATOR network reporting guideline platform [Internet]. The UK EQUATOR Centre; 2025. Available from: https:/resources.equator-network.org/reporting-guidelines/strobe/strobe-checklist.docx
